# *Bordetella pertussis* Infection in South African HIV-Infected and HIV-Uninfected Mother–Infant Dyads: A Longitudinal Cohort Study

**DOI:** 10.1093/cid/ciw527

**Published:** 2016-11-02

**Authors:** Marta C. Nunes, Sarah Downs, Stephanie Jones, Nadia van Niekerk, Clare L. Cutland, Shabir A. Madhi

**Affiliations:** 1 Department of Science and Technology/National Research Foundation, Vaccine Preventable Diseases; 2 Respiratory and Meningeal Pathogens Research Unit, Medical Research Council, University of the Witwatersrand; 3 National Institute for Communicable Diseases, National Health Laboratory Service, Centre for Vaccines and Immunology, Johannesburg, South Africa

**Keywords:** *Bordetella pertussis*, HIV infection, mothers, infants

## Abstract

***Background.*** There is a paucity of data regarding the burden of *Bordetella pertussis* in African women and young infants, and particularly the impact of maternal human immunodeficiency virus (HIV) infection thereon. We performed a retrospective analysis of respiratory illness samples from longitudinal cohorts of HIV-uninfected and HIV-infected women and their infants to evaluate the burden of pertussis illness in a black-African community.

***Methods.*** The women were followed up for respiratory illness from midpregnancy and together with their infants until 24 weeks postpartum. Respiratory samples obtained at the time of illness visits were tested for *B. pertussis* by polymerase chain reaction (PCR).

***Results.*** The study included 194 HIV-infected and 1060 HIV-uninfected women, and 188 and 1028 infant offspring, respectively. There were 7 PCR-confirmed pertussis cases in the HIV-exposed infants and 30 in HIV-unexposed infants (7.4 vs 5.5 episodes per 1000 infant-months; *P* = .47), at a mean age of 70.9 days. All infant pertussis cases had a history of cough (mean duration, 6.3 days). Six of 17 (35.3%) pertussis-confirmed cases in infants <2 months of age were admitted to hospital within 21 days of *B. pertussis* detection, whereas none of the 20 cases ≥2 months of age required hospitalization. Ten PCR-positive pertussis-associated illnesses were detected in HIV-infected women compared with 32 in the HIV-uninfected women (6.8 vs 3.9 episodes per 1000 person-months; *P* = .12).

***Conclusions.*** *Bordetella pertussis* identification was common among young infants with respiratory illness, most of whom were too young to be fully protected through direct vaccination. Vaccination of pregnant women might be a valuable strategy in a setting such us ours to prevent *B. pertussis–*associated illness in women and their young infants.

Whooping cough, a highly contagious acute respiratory tract infection caused by the bacterium *Bordetella pertussis*, is a common vaccine-preventable disease. Despite the significant decrease in the burden of disease due to high immunization rates, substantial numbers of pertussis cases are still recorded [1, 2]. Young infants, especially during the first 2 months of life before receipt of pertussis vaccines, are more susceptible to pertussis-associated complications, including hospitalization and death [3–5].

While the classical presentation of pertussis illness is well recognized by uncontrollable coughing paroxysms, atypical milder pertussis disease may occur in individuals with primed immune system either by vaccination or previous infection [6, 7]. Atypical cases generally are not reported, but may significantly contribute to the transmission of *B. pertussis* in the community [8]. Most studies on the burden of pertussis have relied on hospitalized cases or on reporting by general practitioners, which reflects the incidence of severe pertussis illness but likely underestimates the overall incidence of pertussis [1, 4]. Seroprevalence studies are useful to estimate the circulation of *B. pertussis* at the community level, but are influenced by the immunization coverage of the population and are imprecise to estimate the time of infection [9]. To capture the full spectrum of pertussis infection, individuals presenting with severe and less severe respiratory symptoms should be investigated.

The epidemiology of pertussis illness in Africa has not been well described, especially of nonsevere disease in a population with high coverage of acellular pertussis vaccine during childhood [10]. Furthermore, the impact of HIV infection in women of childbearing age and/or in utero infant HIV exposure on the burden of pertussis warrants evaluation.

The aim of this study was to estimate the incidence of pertussis illness in HIV-infected and HIV-uninfected mothers from midpregnancy and together with their live births until 24 weeks postpartum.

## SUBJECTS AND METHODS

### Study Population

South Africa transitioned from whole-cell pertussis to acellular pertussis vaccine in April 2009. A pentavalent vaccine containing diphtheria toxoid, tetanus toxoid, acellular pertussis, trivalent inactivated polio vaccine, and *Haemophilus influenzae* type b conjugate vaccine (DTaP-IPV/HibCV; Pentaxim, Sanofi-Pasteur, Lyon, France) was used to immunize children at 6, 10, and 14 weeks of age and a booster dose was given at 18 months as part of the public immunization program. South Africa is the only country in sub-Saharan Africa currently using an acellular pertussis–containing vaccine in its public immunization program.

Confirmed HIV-uninfected and HIV-infected pregnant women in the second or third trimester were independently enrolled into 2 randomized, double-blind, placebo-controlled trials of trivalent inactivated influenza vaccine (IIV3) in 2011 as described previously [11]. Participants were residents of Soweto, a large black-African urban settlement in the outskirts of Johannesburg, South Africa. Enrollment occurred between 3 March and 4 August 2011, and women were randomized 1:1 to receive the influenza vaccine recommended by the World Health Organization for the Southern Hemisphere in 2011 or sterile 0.9% normal saline solution as placebo. Women were followed up for any respiratory illness from the time of enrollment through pregnancy to 24 weeks postpartum, and their infants from the time of birth to 24 weeks of age. This report describes the illness episodes associated with pertussis infection in maternal and infant participants of the IIV3 trial.

### Sample Collection and Testing

Active surveillance for respiratory illness was done by weekly contact of the study participants throughout the study period. Respiratory specimens (nasopharyngeal aspirate in infants and oropharyngeal plus flocked nasopharyngeal swabs in the women) were collected when study participants (1) attended the study center for any unsolicited respiratory illness; (2) were hospitalized for acute cardiopulmonary illness at the single public hospital serving the study population; and (3) were identified through weekly home visits as having signs and symptoms of respiratory illness (in the women these included: (1) fever or history of chills, rigors or feeling feverish; and (2) presence of cough or sore throat or pharyngitis; or (3) presence of myalgia, arthralgia or headache; or (4) presence of dyspnea, breathing difficulty or chest pain when breathing; and in the infants: (1) axillary temperature ≥37.8°C or mother’s perception that the infant was feverish, coupled with at least one sign or symptom of acute respiratory infection within the past 72 hours, or (2) at least two signs or symptoms of acute respiratory illness within the past 72 hours which included: respiratory rate of ≥60 and ≥50 breaths per minute in infant 0–2 months and 2–6 months of age, respectively; difficulty breathing, cough, wheezing, runny or congested nose, cyanosis or oxygen saturation <90% on room air, chest wall in-drawing, grunting on expiration and pus draining from either ear) and were requested to attend the study centre.

Respiratory specimens were placed in universal transport medium (UTM; Copan, Brescia, Italy) and transported to the Respiratory and Meningeal Pathogens Research Unit laboratory where all samples collected were immediately tested by qualitative 2-step real-time reverse-transcription polymerase chain reaction (PCR) assay for influenza; the remaining complementary DNA and UTM specimens were stored at −20°C and −70°C, respectively. All archived cDNA samples independent of clinical presentation were tested for the presence of the multicopy pertussis insertion sequence (IS) *481* using a modified protocol to the one described by Tatti et al [12]; if IS*481* cycle threshold (Ct) values were ≤40, total nucleic acids were extracted from the corresponding achieved UTM specimen using a NucliSENS easyMAG (bioMérieux) platform and retested by real-time PCR for IS*481* and in a duplex reaction for hIS*1001* and pIS*1001* with an annealing temperature of 57°C, and in a singleplex reaction for the pertussis toxin subunit S1 (*ptxS1*) with an annealing temperature of 60°C. Primers and probes used are listed in Supplementary Table 1. Internal controls were included to check the integrity of the samples and efficiency of the extraction step and to detect the presence of PCR inhibitors. Positive controls were included in each experiment. We followed the criteria in Tatti et al applied to our second PCR to consider a sample PCR positive for *B. pertussis* (Supplementary Table 2); specimens with an intermediate PCR result were deemed as nonpositive in the current analysis [12]. A pertussis case was defined as a positive PCR result on respiratory specimen independent of clinical presentation.

### Clinical Information

At the time of specimen collection, detailed clinical information was recorded in study-specific forms. The presence and duration of the following signs and symptoms were recorded from the infants: fever (≥37.8°C axillary temperature and/ or mother's perception that infant was feverish), history of apnea, tachypnea, difficult breathing, coughing, wheezing, runny or blocked nose, cyanosis, chest wall indrawing, grunting on expiration, and otorrhea; and for the mothers: fever (≥38°C on oral measurements and/or feeling feverish), chills/rigors, cough, sore throat, pharyngitis, muscle or joint aches, headache, feeling short of breath, difficulty breathing, and chest pain while breathing. In addition, other relevant symptoms were recorded as free text. The total duration of cough in the pertussis cases was ascertained retrospectively using information collected during the weekly contacts. For hospitalized cases, complete hospital records were available. Immunization status of the infants was abstracted from the individual official vaccination card.

### Statistical Analysis

Categorical variables were described as proportions and compared by χ^2^ or Fisher exact test; continuous variables were represented as mean or median and compared by Student *t* test or Mann–Whitney test. Incidence was calculated as density incidence using person-time as denominator; 95% confidence intervals (CIs) for incidence estimates were calculated using a Poisson distribution. Follow-up was censored at last contact (if infant was <175 days old), death (if it occurred before infant was 175 days old), when infant was 175 days of age, or at the first pertussis PCR–positive episode, whichever came first. Logistic regression analysis was performed to assess potential risk factors for pertussis infection in HIV-infected women. *P* values <.05 were considered significant. Study data were collected and managed using Research Electronic Data Capture [13]. Analyses were performed using Stata software, version 13.1 (StataCorp, College Station, Texas).

### Ethical Considerations

The initial 2 trials and subsequent analysis of study participants and samples were approved by the Human Research Ethics Committee of the University of the Witwatersrand (HREC numbers 101106 and 101107) and conducted in accordance with Good Clinical Practice guidelines. Mothers provided written informed consent for themselves and their infants. The original trials were registered on ClinicalTrials.gov (NCT01306669 and NCT01306682).

## RESULTS

### Characteristics of the Study Population

One hundred ninety-four HIV-infected and 1060 HIV-uninfected pregnant women participated in the study. HIV-infected women were older than HIV-uninfected women (28.2 vs 26.2 years; *P* < .001). Women in both groups had similar gestational age at enrollment and were followed up for the same amount of time (8 months) (Table 1). Two women in each group died during the study period, none of which were associated with pertussis. One hundred eighty-eight infants born to HIV-infected mothers and 1028 infants born to HIV-uninfected mothers participated in the study. A higher percentage of HIV-exposed compared with HIV-unexposed births occurred before 37 weeks of gestational age (13.8% vs 6.8%; *P* = .001), and the mean follow-up time was shorter in HIV-exposed than in HIV-unexposed infants (5.2 vs 5.4 months; *P* = .001). Fourteen HIV-exposed (7.5%) and 18 (1.8%) HIV-unexposed infants died during the study period (Table 1). Six HIV-exposed infants discontinued the study and 12 died before an HIV test was performed, 18 infants did not have an HIV PCR result available, 1 (0.7%) infant was HIV PCR positive, and the HIV PCR was nonreactive in the remaining 151 (99.3%) infants.

**Table 1.** Demographic and Clinical Characteristics of the Study Participants According to Human Immunodeficiency Virus Status

Characteristic	HIV-Infected (n = 194)	HIV-Uninfected (n = 1060)	*P* Value
Women			
Age at enrollment, y, mean (SD)	28.2 (5.1)	26.2 (5.3)	<.001
Gestational age at enrollment, wk, mean (SD)	27.3 (3.8)	26.9 (4.3)	.205
Follow-up time, mo, mean (SD)	7.8 (1.5)	8.0 (1.8)	.328
Deaths	2 (1.0)	2 (0.19)	.056
CD4^+^ count at enrollment, cells/µL, mean (SD)^a^	446.7 (219.8)	…	…
Women at enrollment with HIV RNA load ≤40 copies/mL^b^	43 (22.8)	…	…
Women at enrollment on antiretroviral therapy^c^	153 (78.9)	…	…
	HIV-Exposed (n = 188)	HIV-Unexposed (n = 1028)	
Infants			
Preterm birth^d^	26 (13.8)	70 (6.8)	.001
Males	96 (51.6)	534 (52.0)	.923
Follow-up time, mo, mean (SD)	5.2 (1.3)	5.4 (0.9)	.001
Deaths	14 (7.5)	18 (1.8)	<.001

Data are presented as No. (%) unless otherwise indicated.

Abbreviations: HIV, human immunodeficiency virus; SD, standard deviation.

^a^ One hundred ninety-one women with available information.

^b^ One hundred eighty-nine women with available information.

^c^ Includes women on prevention of mother-to-child HIV transmission–specific antiretroviral therapy and participants on highly active antiretroviral treatment.

^d^ Births occurred before 37 weeks of gestational age.

### Incidence of PCR-Positive Pertussis Cases in the Infants

A total of 463 respiratory specimens were collected from 147 HIV-exposed infants, of which 428 (92.2%) were available for PCR testing for pertussis. Seven HIV-exposed infants tested pertussis PCR positive and the estimated incidence was 7.4 (95% CI, 3.5–15.5) episodes per 1000 infant-months (Table 2). Seven hundred six HIV-unexposed infants had at least 1 illness visit during the first 24 weeks of life and 1772 specimens were collected; of these, 1659 (93.6%) were tested for pertussis. Thirty HIV-unexposed infants had a PCR-positive pertussis episode, yielding an incidence of 5.5 (95% CI, 3.8–7.8) episodes per 1000 infant-months (incidence rate ratio, 1.4 [95% CI, .5–3.1] HIV-exposed vs HIV unexposed) (Table 2).

**Table 2.** Pertussis Polymerase Chain Reaction–Positive Incidence Rates According to Human Immunodeficiency Virus Status

Category	No. of Cases	Person-time, mo	Incidence per 1000 Person-months (95% CI)	Attack Rate per 1000 Participants (95% CI)
Infants				
HIV-exposed overall	7	948.5	7.4 (3.5–15.5)	37.2 (15.1–75.2)
HIV-unexposed overall	30	5488.5	5.5 (3.8–7.8)	29.2 (19.8–41.4)
* P* value			.474	.562
* P* value adjusted^a^			.520	.605
HIV-exposed 0–90 d of age	6	518.7	11.6 (5.2–25.8)	31.9 (11.8–68.2)
HIV-unexposed 0–90 d of age	19	2945.5	6.5 (4.1–10.1)	18.5 (11.2–28.7)
* P* value			.212	.243
* P* value adjusted^a^			.215	.242
HIV-exposed >90 d of age	1	444.9	2.2 (.32–16.0)	5.7 (.14–31.2)
HIV-unexposed >90 d of age	11	2590.7	4.2 (2.4–7.7)	11.1 (5.6–19.8)
* P* value			.543	.522
* P* value adjusted^a^			.485	.467
Overall	37	6437.0	5.8 (4.2–7.9)	30.4 (21.5–41.7)
0–90 d of age	25	3464.2	7.2 (4.9–10.7)	20.6 (13.3–30.2)
>90 d of age	12	3035.6	4.0 (2.2–7.0)	10.3 (5.3–17.9)
* P* value^b^			.086	.042
Women				
HIV-infected overall	10	1469.1	6.8 (3.7–12.7)	51.6 (26.0–92.8)
HIV-uninfected overall	32	8261.5	3.9 (2.7–5.5)	30.2 (20.7–42.4)
* P* value			.120	.140
* P* value adjusted^c^			.086	.100

Abbreviations: CI, confidence interval; HIV, human immunodeficiency virus.

^a^ *P* value adjusted for preterm births.

^b^ *P* value comparing infants aged 0–90 days old vs >90 days old overall.

^c^ *P* value adjusted for age at enrollment.

Seventy percent of the pertussis cases were male (odds ratio, 2.2 [95% CI, 1.1–4.6]), and the mean age at pertussis infection was 70.9 days (Table 3). Eleven (29.7%), 6 (16.2%), 8 (21.6%), and 12 (32.4%) cases were ≤30 days, 31–60 days, 61–90 days, and >90 days of age, respectively, with no differences between HIV-exposed and HIV-unexposed infants. Thirteen infants were too young (<6 weeks of age) to have received any pertussis-containing vaccine at the time of their pertussis episode. Of the remaining 24 pertussis cases, immunization records were available for 23 (95.8%); of the 5 cases eligible to have received 2 vaccine doses, 3 received both doses but the second dose was received <10 days before the onset of the pertussis episode. Of the 11 cases old enough to receive 3 vaccine doses, 5 were fully vaccinated, 1 of whom received the third dose during the week when testing pertussis PCR positive (Table 4). Overall, 88.8% of pertussis cases had not completed their primary series of DTaP-IPV/HibCV (ie, at least 14 days after the third dose).

**Table 3.** Characteristics and Clinical Presentation of Pertussis Cases in Human Immunodeficiency Virus (HIV)-Exposed and HIV-Unexposed Infants

Characteristic	HIV-Exposed (n = 7)	HIV-Unexposed (n = 30)	Overall (N = 37)
Male	5 (71.4)	21 (70.0)	26 (70.3)
Age at pertussis detection, d, mean (SD)	67.3 (49.3)	71.8 (46.1)	70.9 (46.0)
Preterm birth	1 (14.3)	3 (10.0)	4 (10.8)
Antibiotic treatment during the pertussis episode	4 (57.1)	15 (50.0)	19 (51.4)
Macrolides	0	5 (16.7)	5 (13.5)
Hospitalized within 21 d of specimen collection	2 (28.6)	4 (13.3)	6 (16.2)
Pertussis-associated death	0	1 (3.3)	0
Symptoms presented during the pertussis episode			
Cough	7 (100)	30 (100.0)	37 (100)
Cough duration ≥14 d	1 (14.3)	7 (23.3)	8 (21.6)
Fever	2 (28.6)	7 (23.3)	9 (24.3)
Apnea	1 (14.3)	2 (6.7)	3 (8.1)
Tachypnea	3 (42.9)	5 (16.7)	8 (21.6)
Difficulty breathing	57.1 (4)^a^	20.0 (6)^a^	10 (27.0)
Wheezing	3 (42.9)	5 (16.7)	8 (21.6)
Runny or blocked nose	5 (71.4)	22 (73.3)	27 (73.0)
Cyanosis	0	1 (3.3)	1 (2.7)
Chest wall indrawing	2 (28.6)	3 (10.0)	5 (13.5)
Grunting on expiration	0	2 (6.7)	2 (5.4)
Otorrhea	0	0	0
Vomiting	2 (28.6)	4 (13.3)	6 (16.2)
Paroxysmal cough with or without whoop^b^	1 (14.3)	4 (13.3)	6 (16.2)
Lethargy^b^	0	1 (3.3)	1 (2.7)
Poor feeding^b^	2 (28.6)	3 (10.0)	5 (13.5)
Pulmonary signs	2 (28.6)	11 (36.7)	13 (35.1)

Data are presented as No. (%) unless otherwise indicated.

Abbreviations: HIV, human immunodeficiency virus; SD, standard deviation.

^a^ HIV-exposed vs HIV-unexposed *P* value = .046.

^b^ Information collected from reviewing hospital records.

**Table 4.** Immunization Status of Infants With Polymerase Chain Reaction–Positive Pertussis

Age	Total No. of Cases	Information Available	DTaP Dose 1 (6 wk)	DTaP Dose 2 (10 wk)	DTaP Dose 3 (14 wk)
0–6 wk	13		0	0	0
7–10 wk	8	7	6^a^	0	0
11–14 wk	5	5	5	3^b^	0
>14 wk	11	11	9	6	6^c,d^

Abbreviation: DTaP, pentavalent vaccine containing diphtheria toxoid, tetanus toxoid, acellular pertussis, trivalent inactivated polio vaccine, and *Haemophilus influenzae* type b conjugate vaccine.

^a^ Two pertussis cases were ≤12 days postvaccination.

^b^ The 3 pertussis cases were ≤10 days postvaccination.

^c^ One pertussis case was 7 days postvaccination.

^d^ One pertussis case missed the 10-week dose.

All 37 infant PCR-positive pertussis cases had a history of cough; however, only in 8 (21.6%) did the cough persist for at least 14 days. The mean duration of cough was 6.3 (standard deviation [SD], 7.1) days, and 73% had rhinorrhea or blocked nose. The classical paroxysmal cough was noted in 6 (16.2%) pertussis cases, and 13 (35.1%) pertussis episodes were associated with lower respiratory tract infection. Comparing the frequency of the clinical signs and symptoms associated with pertussis PCR positivity between HIV-exposed and HIV-unexposed infants, no differences were detected except that difficulty breathing was more common in HIV-exposed infants (57.1% vs 20.0%; *P* = .046; Table 4). Antibiotics were prescribed during the pertussis episodes to 19 (51.4%) cases, including only 5 (13.5%) who received a macrolide.

Six of 17 (35.3%) infants <2 months of age were admitted to hospital within 21 days of pertussis detection, compared with 0% of the 20 infants >2 months of age (relative risk, 15.2; *P* = .06). Four of the 6 hospitalized cases were diagnosed with suspected pertussis by the attending physician, with 3 also being tested for *B. pertussis* through their routine care. Of the physician clinically suspected cases, 1 was associated with laryngotracheobronchitis, 1 with neonatal sepsis, and 2 with pneumonia; of the 2 other admissions, 1 was diagnosed as pneumonia and a 39-day-old infant presented with apnea. One (16.6%) of the hospitalized HIV-unexposed infants, who was diagnosed with suspected pertussis associated with pneumonia, died in the intensive care unit at 61 days of age; he was treated with erythromycin/ampicillin/cefotaxime, and his vaccination status was unknown.

### Incidence of PCR-Positive Pertussis Cases in the Women

One hundred forty-five HIV-infected women had at least 1 illness visit from enrollment to 24 weeks postpartum; 476 respiratory specimens were collected and, of these, 429 (90.1%) were tested by PCR for *B. pertussis*. Ten HIV-infected women tested pertussis PCR positive, and the estimated incidence was 6.8 (95% CI, 3.7–12.7) episodes per 1000 person-months. In the HIV-uninfected cohort, 1491 specimens were collected from 646 women; of these, 1298 (87.1%; *P* = .06 compared with HIV-infected women) were tested for *B. pertussis*. HIV-uninfected women had a lower incidence of PCR-positive pertussis (32 cases) of 3.9 (95% CI, 2.7–5.5) episodes per 1000 person-months compared with HIV-infected women, albeit not significant (*P* = .12; Table 2). For both HIV-infected and HIV-uninfected women, the incidence of pertussis infection was similar compared to their infants (7.4 [*P* = .89] and 5.5 [*P* = .45] per 1000 infant-months, respectively).

The clinical presentation of the women with PCR-positive pertussis included 85.7% with cough (14.3% in whom cough persisted ≥14 days) with a mean duration of 8.6 (SD, 8.4) days; and 64.3% with rhinorrhea or blocked nose. Comparing the frequency of clinical signs and symptoms in the pertussis cases between HIV-infected and HIV-uninfected women, no significant differences were detected, except that chest pain was recorded in a higher percentage of HIV-infected women (40.0% vs 6.3%; *P* = .010). Antibiotics were also prescribed more frequently to HIV-infected women during the pertussis episode (70.0% vs 25.0%; *P* = .010). None of the women were prescribed macrolides or hospitalized (Table 5).

**Table 5.** Characteristics and Clinical Presentation of Pertussis Cases in Human Immunodeficiency Virus (HIV)-Infected and HIV-Uninfected Women

Characteristic	HIV-Infected (n = 10)	HIV-Uninfected (n = 32)	Overall (N = 42)
Antibiotic treatment during the pertussis episode, No. (%)	7 (70.0)^a^	8 (25.0)^a^	15 (35.7)
Symptoms presented during the pertussis episode, No. (%)			
Cough	9 (90.0)	27 (84.4)	36 (85.7)
Cough duration ≥14 d	2 (20.0)	4 (12.5)	6 (14.3)
Fever	0	4 (12.5)	4 (9.5)
Runny or blocked nose	6 (60.0)	21 (65.6)	27 (64.3)
Chills/rigors	1 (10.0)	7 (21.9)	8 (19.1)
Sore throat	2 (20.0)	12 (37.5)	14 (33.3)
Pharyngitis	1 (10.0)	1 (3.1)	2 (4.8)
Muscle or joint aches	1 (10.0)	4 (12.5)	5 (11.9)
Headaches	7 (70.0)	15 (46.9)	22 (52.4)
Short of breath	1 (10.0)	2 (6.3)	3 (7.1)
Difficulty breathing	1 (10.0)	1 (3.1)	2 (4.8)
Chest pain while breathing	4 (40.0)^a^	2 (6.3)^a^	6 (14.3)

Abbreviation: HIV, human immunodeficiency virus.

^a^ HIV-infected vs HIV-uninfected *P* value = .010.

In HIV-infected women, CD4^+^ cell count, HIV RNA load, and being on antiretroviral therapy at study enrollment were not associated with pertussis PCR positivity during the study period (Supplementary Table 3).

## DISCUSSION

Our study reported a high incidence of pertussis PCR positivity in infants ≤24 weeks of age (5–7 episodes per 1000 infant-months; or 29–37 per 1000 infants) and in the mothers of these children (4–7 episodes per 1000 person-months; or 30–52 per 1000 persons). The incidence of pertussis infection has not been described before in South Africa, and we are unaware of any other published report that used a similar approach to ours elsewhere, hence limiting direct comparisons to others.

Pertussis infection, especially in adolescents and adults, can range from classic pertussis presentation to milder symptoms with only rhinitis or present even without cough [14]. In our study, we were able to capture the entire spectrum of infection as the collection and testing of samples did not follow a predefined algorithm, we undertook active weekly surveillance for presence of respiratory symptoms, and we had a low threshold of investigating unsolicited respiratory illness visits irrespective of symptom severity. In conjunction with using a highly sensitive and specific PCR protocol [12], the cumulative incidence presented here for both infants and mothers likely reflects the overall incidence of pertussis disease in the target population; the majority of illness was mild in infants >2 months of age and in women.

The incidence of pertussis in both high-income and lower-middle-income countries is probably significantly higher than currently reported by either medical notifications or hospitalizations [15]. No surveillance system is perfect to register all cases. Population-based seroprevalence studies are valuable to estimate the incidence of pertussis infection, although not necessarily clinically significant pertussis disease, and can also be used to compare within and between countries with different diagnostic practices [16, 17]. Nevertheless, qualitative serological tests are known to have low specificity, mainly in recently and frequently immunized populations [18]. Estimated rates of pertussis infection generated from seroprevalence studies in both children and adults have been described as 100–1000 times higher than the official reported disease incidences [19–21]. Kretzschmar et al, applying a method based on the entire antibody distribution, estimated that the seroincidence in 5 European countries was between 1% and 6% per annum [16]. Furthermore, a recent study combining the estimated incidence of infection with notification data and symptomatic probabilities estimated the incidence of symptomatic pertussis in the Netherlands to be approximately 100 cases per 10 000 (1%) [22]. The attack rates of PCR-confirmed pertussis infection identified in our study among the women (3.0%–5.2%) and infants (2.9%–3.7%) were similar to the published seroincidence estimates, including a recent study from The Gambia where in 2008, 6% of the population between 2 and 90 years of age had antibody concentrations indicative of recent pertussis infection [9].

Most of the pertussis cases in our study, both in infants (except those <2 months of age) and mothers, were nonsevere but resulted in a medical illness visit within the context of our clinical trial. Using our approach, we were also able to describe the disease and calculate incidence for infants independent of their vaccination status. Even though all infants had cough, very few presented the classical picture of vomiting, whooping, and paroxysmal coughing. Eighty-nine percent of the infants with pertussis illness had not completed their primary series of DTaP-IPV/HibCV. In our study, boys were at higher risk of pertussis infections than girls, which is in contrast to that reported from medical notification studies [23], although the higher pertussis incidence in females was mainly seen among adult cases [24]. Although approximately 50% of the pertussis PCR–positive cases were >2 months, using hospitalization as a proxy for severity, the most severe disease was manifest in infants <2 months of age, among whom one-third of infections resulted in hospitalization. This would be the spectrum of disease that maternal immunization against pertussis will need to be most effective in preventing.

A study from South Africa in HIV-unexposed and HIV-exposed infants indicated that 3–4 doses of an acellular pertussis–containing vaccine are required to achieve good humoral responses, suggesting that the period of suboptimal protection against pertussis may be at least 3–4 months of age in our setting [25]. Lower antibody titers to pertussis prevaccination have been detected in HIV-exposed compared with HIV-unexposed infants; conversely, HIV-exposed infants display stronger pertussis vaccine responses following primary series of vaccination [25–27]. Although there is no established correlate of protection against pertussis, it could be speculated that the lower pertussis antibody levels among HIV-exposed infants prevaccination could increase their susceptibility to pertussis disease. In our study, although there was a higher point estimate for pertussis infection among HIV-exposed compared with HIV-unexposed infants, this was not statistically significant and was independent of age and vaccination status. HIV-infected women also had a nonsignificantly higher incidence compared with HIV-uninfected women. Unfortunately, the HIV-infected cohort was also much smaller than the HIV-uninfected cohort, limiting the study to demonstrate an increased risk in the HIV-infected group.

Other limitations include that the study was conducted during a specific period of the year (mainly spanning the fall-winter-spring seasons) and only for a single year, which could influence the incidence results, as *B. pertussis* circulation displays seasonal and long-term periodicity [28]. Also, as the endpoint of the initial study was not pertussis illness, we did not specifically record the common pertussis clinical manifestations, which could have been omitted at the time of sample collection. Nevertheless, if a participant had paroxysmal cough or inspiratory whoop, this information would likely have been recorded by the study doctor. We were able to test 93% and 87% of all the specimens collected from the infants and the mothers respectively; the samples unavailable for testing were missing at random and would probably not affect the incidence estimates.

The antimicrobial treatment for pertussis is macrolides; however, in our study no women received macrolides and only 14% of the infant cases received a macrolide. Treatment of pertussis cases with macrolides has been shown to reduce the duration of shedding of *B. pertussis.* The lack of recognition of mild pertussis, and consequent absence of targeted macrolide treatment, reinforce the problem of unrecognized cases that could affect the transmission dynamics of *B. pertussis* to infants too young to be vaccinated and who have high complication rates [4, 29]. Also, the sustained circulation of *B. pertussis* through mild or asymptomatic carriers enables the pathogen to evolve and escape immune pressure [30]. Our study corroborates the need for pertussis vaccination of pregnant women, which could protect the mothers themselves, as well as their offspring. The cost-effectiveness of this strategy, however, requires further interrogation before it is implementable in resource-limited countries such as South Africa.

## Supplementary Data

Supplementary materials are available at http://cid.oxfordjournals.org. Consisting of data provided by the author to benefit the reader, the posted materials are not copyedited and are the sole responsibility of the author, so questions or comments should be addressed to the author.

Supplementary Data
