# Supplementary material for: Bordetella pertussis Infection in South African HIV-Infected and HIV-Uninfected Mother–Infant Dyads: A Longitudinal Cohort Study
Source: Clin Infect Dis. 2016 Nov 2;63(Suppl 4):S174–80. doi: 10.1093/cid/ciw527 (PMC5106617; doi:10.1093/cid/ciw527)
Supplement: Supplementary Data [file supp_63_suppl-4_S174__index.html]

Supplementary Data 

# *Bordetella pertussis* Infection in South African HIV-Infected and HIV-Uninfected Mother–Infant Dyads: A Longitudinal Cohort Study

## Supplementary Data

Supplementary Data

- Supplementary Data - Pdf file
