# Supplementary material for: Bordetella pertussis Infection in South African HIV-Infected and HIV-Uninfected Mother–Infant Dyads: A Longitudinal Cohort Study
Source: Clin Infect Dis. 2016 Nov 2;63(Suppl 4):S174–80. doi: 10.1093/cid/ciw527 (PMC5106617; doi:10.1093/cid/ciw527)
Supplement: Supplementary Data [file supp_ciw527_ciw527supp.pdf]

Supplementary Table 1. Primers and Probes sequences

Gene Name	Sequence (5'-3')
IS481	For 5'-CAAGGCCGAACGCTTCAT-3'<br>Rev 5'-GAGTTCTGGTAGGTGTGAGCGTAA-3'<br>Probe 5'-NED-CAGTCGGCCTTGCGTGAGTGGG-MGB-3'
PtxS	For 5'-CGCCAGCTCGTACTTC-3'<br>Rev 5'-GATACGGCCGGCATT-3'<br>Probe 5'-VIC-AATACGTCGACACTTATGGCGA-MGB-3'
hIS1001	For 5'- GGCGACAGCGAGACAGAATC -3'<br>Rev 5'- GCCGCCTTGGCTCACTT -3'<br>Probe 5'-VIC- CGTGCAGATAGGCTTTTAGCTTGAGCGC–MGB-3'
pIS1001	For 5'- TCGAACGCGTGGAATGG -3'<br>Rev 5'- GGCCGTTGGCTTCAAATAGA -3'<br>Probe 5'-FAM- AGACCCAGGGCGCACGCTGTC-MGB3'
Human *rnaseP*	For 5'-AGATTTGGACCTGCGAGCG-3'<br>Rev 5'-GAGCGGCTGTCTCCACAAGT-3'<br>Probe 5'-FAM-TTCTGACCTGAAGGCTCTGCGCG-MGB3'
Human *GAPDH*	For 5'- GCTCCTCCTGTTCGACAGTCA -3'<br>Rev 5'- ACCTTCCCCATGGTGTCTGA -3'<br>Probe 5'-NED-CGTCGCCAGCCGAGCCACA -MGB3'

Supplementary Table 2. PCR results interpretation

Targets				Interpretation
IS481	p*IS1001*	h*IS1001*	*ptx* S1	
CT<35	-	-	+ or -	*B. pertussis*
CT ≤35-<40	-	-	+	*B. pertussis*
CT ≤35-<40	-	-	-	indeterminate
Positive	-	+	-	*B. holmesii*

Supplementary Table 3. Association of HIV-infection makers and pertussis illness in HIV-infected women

Factor	Pertussis cases	Non-pertussis cases	OR (95% CI); p-value
CD4+ count at enrolment; n (%)			
≥500 cells/mm^3	2/9 (22.2)	59 (32.4)/182	reference
350-<500 cells/mm^3	5/9 (55.6)	58 (31.9)/182	2.54 (0.47, 13.64); 0.276
200-<350 cells/mm^3	2/9 (22.2)	50 (27.5)/182	1.18 (0.16, 8.68); 0.871
<200 cells/mm^3	0/9 (0)	15 (8.2)/182	-
HIV-1 viral load at enrolment; n (%)			
≤40 copies/ml	1/9 (11.1)	138 (76.7)/180	reference
>40 copies/ml	8/9 (88.9)	42 (23.3)/180	2.43 (0.30, 20.03); 0.408
Antiretroviral therapy at enrolment; n (%)			
No Antiretroviral therapy	2/10 (20.0)	39/184 (21.2)	reference
On Antiretroviral therapy	8/10 (80.0)	145/184 (78.8)	1.08 (0.22, 5.27); 0.928

OR: Odds ratio (95% confidence intervals) calculated by regression analysis.
